# Maternal BMI and nutritional status in early pregnancy and its impact on neonatal outcomes at birth in Bangladesh

**DOI:** 10.1186/s12884-019-2571-5

**Published:** 2019-11-11

**Authors:** Bishwajit Bhowmik, Tasnima Siddique, Anindita Majumder, Ibrahimu Mdala, Israt A. Hossain, Zahid Hassan, Ishrat Jahan, Nayla Cristina do V. Moreira, Abdul Alim, Abdul Basit, Graham A. Hitman, Abul Kalam A. Khan, Akhtar Hussain

**Affiliations:** 1 Centre of Global Health Research, Diabetic Association of Bangladesh, Dhaka, 1200 Bangladesh; 2 Institute of Health and Society, Faculty of Medicine, University of Oslo, 0318 Oslo, Norway; 3 Department of Pathology, Ibrahim Medical College, Diabetic Association of Bangladesh, Dhaka, 1200 Bangladesh; 4 Department of Biochemistry & Cell Biology, Bangladesh University of Health Sciences, Dhaka, 1216 Bangladesh; 5 Dept of Physiology and Molecular Biology, Bangladesh University of Health Sciences, Dhaka, 1216 Bangladesh; 6 Maternal and Child Health Training Institute, Dhaka, Azimpur 1205 Bangladesh; 7 Non Communicable Disease Control, DGHS, Mohakhali Dhaka, Bangladesh; 8 Baquai Institute of Diabetology and Endocrinology, Baquai Medical University, Karachi, Pakistan; 9 Blizard Institute, Barts and The London School of Medicine and Dentistry, Queen Mary University of London, 4 Newark Street: London E1 2AT, London, United Kingdom; 10 Faculty of Health Sciences, Nord University, 8049 Bodø, Norway; 11 Faculty of Medicine, Federal University of Ceara (FAMED-UFC), Fortaleza, Ceara 60020-181 Brazil; 12 Centre for Global Health Research, Diabetic Association of Bangladesh, Dhaka, 1000 Bangladesh

**Keywords:** Maternal nutrition, Early pregnancy, Cardio-metabolic status, Newborns at birth

## Abstract

**Background:**

To assess the maternal characteristics and nutritional status according to body mass index (BMI) at 6–14 weeks of gestation and to examine the relationship between maternal nutritional status in early pregnancy and its impact on neonatal birth weight.

**Methods:**

The investigation was conducted from April 2011 to June 2012 in Dhaka, Bangladesh. A total of 498 primigravida pregnant women participated in the study; women with known diabetes or previous gestational diabetes (GDM) were excluded. Maternal demographic details, pregnancy history and anthropometric measurements were obtained from the mother at the recruitment (6–14 weeks), 2nd visit between 24 and 28 week of gestation and 3rd visit at delivery. Cord venous blood samples of newborns (*n* = 138) were collected immediately after delivery for blood glucose, insulin, lipid profile, leptin and micronutrients including serum folate, ferritin, homocysteine, vitamin D, and vitamin B12.

**Results:**

The prevalence at 6–14 weeks of pregnancy of anemia (Hb, < 11 g/dl), vitamin D deficiency (< 30 nmol/l), vitamin B12 deficiency (< 200 pg/ml), high homocysteine level (> 15 μmol/l), folate deficiency (< 3 ng/ml) and iron deficiency (ferritin < 13 ng/ml) were 19.5, 46.4, 15.1, 1.2, 0.4, and 12.7% respectively. GDM was found in 18.4% women. The prevalence of GDM was higher in overweight women (28.1%) than underweight (16.7%) and normal weight women (16.0%: *p* <  0.05). The incidence of low birth weight (LBW) and preterm delivery were 11.6 and 5.8% respectively and was not related to maternal BMI at 6–14 weeks of pregnancy. Maternal height was positively (*p* = 0.02), and homocysteine was negatively associated with neonatal birth weight (*p* = 0.02). In addition, the newborn’s cord serum folate was positively (*p* = 0.03) and cord triglyceride was negatively (*p* = 0.03) associated with neonatal birth weight.

**Conclusion:**

Multiple maternal micronutrient deficiencies were present in early pregnancy. Maternal BMI in early pregnancy was not related to preterm deliveries or LBW. LBW was associated with lower folate, elevated cord triglyceride concentrations of the neonates and mother’s height and increase in maternal homocysteine levels. The data has important implications for pregnancy care in Bangladesh and other similar communities.

## Background

Maternal nutrition plays an important role in placental-fetal growth and development. Maternal undernutrition during pregnancy results in intrauterine growth restriction (IUGR) which is associated with increased perinatal morbidity and mortality. These children have an increased risk for development of metabolic syndrome in adult life [1, 2].

Evidence shows that the animal embryo comprises two group of cells in the early embryonic stage. The inner cell mass which develops as the fetus and the outer cell mass become the placenta. These developmental process is influenced by nutrition and hormones [3, 4]. Maternal undernutrition at the time of conception have shown fewer cells in the inner cell mass in experimental animal studies, which is associated with reduced birth weight and postnatal growth, altered organ/body weight ratios and the development of chronic diseases as type 2 diabetes (T2DM), hypertension (HTN), coronary artery disease (CAD) etc. [5] Evidence has also suggested that disturbances during critical periods of fetal development alter the structure or function of distinct cells, organ systems or homoeostatic pathways. Therefore, the subjects will have an increased risk of developing cardiovascular disease and T2DM in later life [6]. Studies in different time points have found the association between maternal malnutrition in early pregnancy and the occurrence of CAD in the offspring. They have shown that exposure to malnutrition, especially in late gestation, is linked to impaired glucose tolerance, while exposure in early gestation is linked to atherogenic lipid disorders and obesity [7–9].

Low birth weight (LBW) is considered an indicator of undernutrition in utero. Epidemiological studies have shown that it is associated with a wide range of adverse outcomes in later life, including shorter stature, lower cognitive performance, increased risk factors for later chronic non-communicable disease including, T2DM, HTN, CAD, chronic lung and kidney disease [10].

Even though, the obesity level is rising across the globe, yet more than 20 million infants (15.5% of all births) are born each year at LBW (< 2.5 kg). LBW remains a problem focused in developing countries, and half of all LBW babies are born in South and central Asia [11]. Although fetal growth and birth weight are known to be influenced by genetic factors, it is influenced most significantly by mothers’ health and nutritional status before and during pregnancy [12]. In economical transitioning countries, rapid changes in lifestyle and nutrition supply within a short time can create a “mismatch”. A study has shown that adults who had a coronary event had been small at birth and thin at 2 years of age and thereafter put on weight rapidly. This type of growth pattern is also related to insulin resistance in later life [13].

Data from Bangladesh in this issue is still scarce. As dietary patterns vary from country to country, especially regarding vegetarian and non-vegetarian components in Bangladesh and its neighboring country Indi. Data from India may not be directly applicable to people living in Bangladesh [14].

The purpose of the exploratory study was to examine the relationship between maternal nutritional status as assessed by body mass index (BMI) in early pregnancy and its impact on pregnancy outcomes and neonatal birth weight. In addition, we examined the relationship between neonatal birth weight with neonatal metabolic and micronutrients characteristics.

## Methods

### Study design and population

This is one of several sub studies of a prospective multi-center EU FP7 project GIFTS (Genomic and lifestyle predictors of fetal outcome relevant to diabetes and obesity and their relevance to prevention strategies in South Asian peoples) project conducted from April 2011 and June 2012 in Dhaka, Bangladesh. Other GIFTS studies included analysis of other populations in Pakistan [15] and Bangladeshi’s in the UK, a micronutrient intervention study in pregnancy in Dhaka (ISRCTN Number: 83599025), genomic predictors of birthweight and gestational diabetes (GDM) in South Asian populations, analysis of body composition in Bangladeshi children aged 2–4 in whose mothers were exposed to a community mobilization program [16], studies in GDM [17–19] and social and cultural influences of South Asian women during pregnancy [20].

Study participants were recruited from five areas (Azimpur, Lalbag, Hazaribag, Kamrangirchar and Keraniganj) in the Dhaka city, Bangladesh through the monthly household visit by Government Family Welfare Visitors (FWV). These areas are from both urban and semi-urban areas. During these visits, pregnant women were identified by a history of a missed menstrual cycle. Upon confirmation of pregnancy by urine pregnancy test women were invited to visit the Maternal and Child Health Training Institute (MCHTI), a tertiary Government hospital for antenatal care and registration.

A given error margin (α) of 5% with 95% confidence from a population of 100,000 and with a prevalence of undernutrition 30% [21], the required sample size is 322 (two-sided test).

Pregnant women were recruited in first trimester of their pregnancy (≤14-week gestation). Women who were primigravida, singleton, conceiving without treatment of fertility and were willing to participate in the study were included after written consent (*n* = 498). Women who had a history of T2DM, or history of GDM or pregnancy induced HTN were excluded from this study.

Date of last menstrual period and ultra-sonographic examination was performed at the time of recruitment to register the duration of gestation. Ultra-sonographic estimation was used as the criterion in case of disparity.

**Fig. 1:**
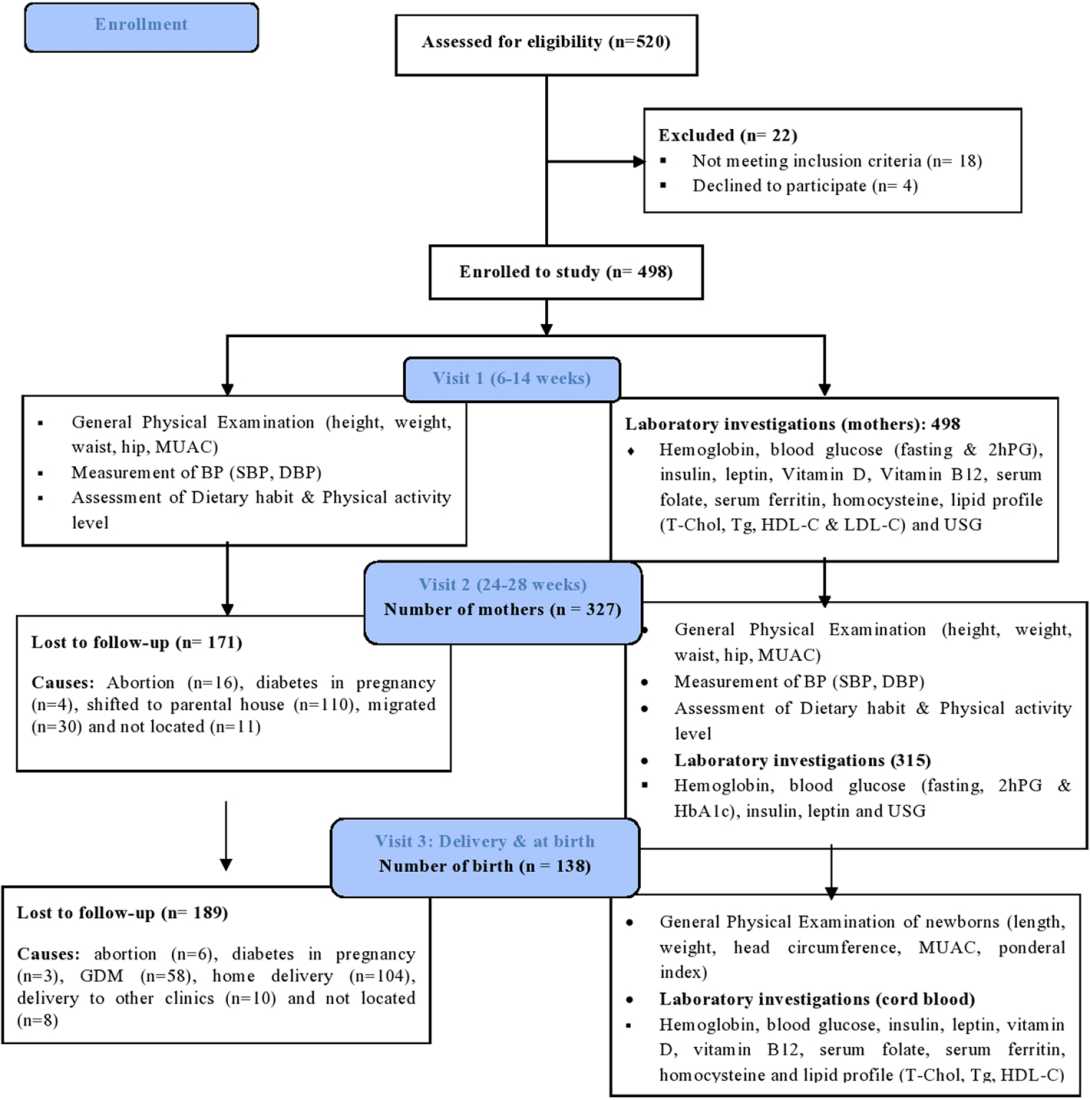
Study flow diagram

All records from each participant were kept during the study period (1st visit in first trimester at the time of registration (between 6 and 14 weeks, *n* = 498), 2nd visit between 24 and 28 week of gestation (*n* = 327) and 3rd visit at delivery (*n* = 138). Figure 1 is the study flow diagram detailing the dropouts at each stage of the process. Each study visit was performed after an overnight fast of 8–12 h.

GDM was diagnosed following a slight modification of International Association of Diabetes and Pregnancy Study Group (IADPSG) criteria in the 2nd trimester (24–28 weeks of gestation). Diagnosis was made by fasting and 2 hours after ingesting 75 g glucose. This was done to avoid high dropout rate because of 3 blood measures in pregnancy. GDM was defined as a fasting plasma glucose (FPG) ≥5.1 mmol/L or plasma glucose ≥8.5 mmol/L 2 hours after ingesting 75 g glucose (2hPG) [15]. Diabetes in pregnancy was defined as a FPG ≥7.0 mmol/L or 2hPG ≥11.1 mmol/L [22]. Women diagnosed with GDM and diabetes in pregnancy in visit 2 were not followed-up to birth as they gave birth in Bangladesh Institute of Research and Rehabilitation in Diabetes, Endocrine and Metabolic Disorders (BIRDEM 2), a specialist Hospital of Diabetic Association of Bangladesh.

### Maternal data collection

Data were collected using questionnaire, physical examination and laboratory investigations. Four trained personnel were involved in data collection. A structured pre-formulated questionnaire was used to collect information on age, place of residence, education, type of occupation, socio-economic status (assessed by total family monthly expenditure), family history of chronic diseases including T2DM, HTN, CAD, and stroke, history of participants’ past illness or any chronic disease, history of addiction, physical activity level, nutritional status, and pregnancy-related history from the mother at the baseline visit. Maternal anthropometry including height, weight, waist, hip and mid upper arm circumference (MUAC) were measured (using standardized measurement protocols) at the time of registration and repeated at the second visit between 24 and 28 weeks’ gestation. BMI was calculated as weight divided by height squared (kg/m^2^). Waist circumference was measured by placing a plastic tape at the midpoint between the lower rib margin and the iliac crest. Similarly, hip was measured by taking the extreme end posteriorly and the symphysis pubis anteriorly. Mid arm circumference (MAC) of the left upper arm, measured at the mid-point between the tip of the shoulder and the tip of the elbow. In addition, blood pressure was measured in right hand in sitting position. Prior to the measurement, 10-min rest was assured and using standard cuffs fitted with sphygmomanometer minimized variation in measurement. Maternal food and nutrient intake were assessed at each trimester through Food Frequency Questionnaires (FFQs). The FFQ used in this study had a food list of 135 items. The FFQ consisted of frequency categories (daily, weekly and monthly). Local household utensils including glasses, spoons, cups and plates were used for estimating the amount of foods and beverages consumed by the respondents. These local utensils acted as visual aids to increase the accuracy of portion size estimations. Food Photo Manual was used, which converts food items of different sizes and composition to gram equivalents. Physical activity levels of participants at work, at home and during leisure time (minutes/day) was recorded following a structured questionnaire used in Bangladesh previously [23].

### Maternal blood sample collection and storage

Blood samples were collected for hemoglobin, lipid profile, circulating serum folate, ferritin, homocysteine, vitamin D (25-Hydroxyvitamin D), and vitamin B12 at 1st visits and glucose (Fasting and 2-h after 75 g oral glucose), insulin and leptin at both visits (1st and 2nd). Serum samples were used for the estimation of serum glucose, lipid profile, insulin, leptin, vitamin D, vitamin B12, folate, ferritin, homocysteine and whole blood used for DNA.

Serum glucose was measured by glucose oxidase (Randox, Laboratories Ltd., UK), serum triglyceride (Tg) by GPO-PAP method (Randox Laboratories Ltd., UK), serum cholesterol (Chol) by enzymatic endpoint method (CHOD-PAP- Randox Laboratories Ltd., UK) and serum high density lipoprotein cholesterol (HDL-C) by differential precipitation, enzymatic colorimetric test (Linear Chemicals, Spain), and low density lipoprotein cholesterol (LDL-C) was estimated by Friedewald’s formula. All serum glucose and lipid profile were measured by using biochemistry auto analyzer (HITACHI 704 Japan), serum insulin, leptin, vitamin D were measured using ELISA kits (DRG Instruments GmbH, Germany). Serum folate, ferritin and vitamin B12 were measured using chemiluminescence immunoassay (Immulite-1000 / Siemens, USA). Serum total homocysteine was measured using Microparticle Enzyme Immunoassay (Abbott AxSYM/ produced by Axis-Shield, Oslo, Norway). Serum was not allowed to be thawed until the assay is performed at the Reference Laboratory in Bangladesh University of Health Sciences (BUHS), Dhaka, Bangladesh. Homeostasis model assessment of insulin resistance (HOMA-IR) was calculated using the method of Matthews et al [24] Intra assay and inter assay coefficient of variations for the measurement of insulin (5.5 and 4.9), B12 (3.1 and 3.74); folate (4.8 and 6.42), homocysteine (3.75 and 1.10) and Vit D (1.7 and 5) were in acceptable ranges.

### Newborn data collection

Newborn characteristics including sex and gestational age at birth were recorded at the time of delivery (*n* = 138). Trained midwives examined all newborns within 24 h of birth. Newborn outcomes of interest included anthropometric characteristics (length, birth weight, head circumference, and mid-upper arm circumference (MUAC)). Newborns’ weight was measured with a digital scale (Model EN-20. MEA/CTN, China) and length was measured using a locally manufactured wooden length board. Head and mid upper arm were measured using a non-stretchable tape.

Umbilical venous cord blood samples of newborns were collected immediately following birth and analyzed for newborn’s serum glucose, insulin, lipids, vitamin D, vitamin B12, serum ferritin, serum folate, homocysteine and leptin. Methods and storage for the analysis of cord blood were same as used for the analysis of maternal blood.

### Statistical analysis

Descriptive statistics in the form of frequencies (n) and percentages (%) were used to describe the maternal BMI at 6–14 weeks of pregnancy, which we categorized into underweight (BMI < 18.5 kg/m^2^), normal weight (BM 18.5–22.9 kg/m^2^) and overweight (BMI ≥23 kg/m^2^) [25]. Similar descriptive statistics were applied to neonatal birth weights, which we categorized as low (< 2500 g) and normal weight (≥2500 g) [26]. We used Analysis of variance (ANOVA) to compare differences of means between mothers with different maternal nutrition status and Independent T-test to compare differences in neonates’ birth weights. Pairwise comparisons between the groups were performed and corrected for multiple testing using Tukey’s HSD. Further, we have used the medians with interquartile range and other related non-parametric tests including Dunn test and Kruskal-Wallis test for skewed variables. Chi-square test was used to test for association between categorical variables. Both simple and multiple linear regression analyses were done for prediction of neonatal birth weight by selected maternal and neonatal parameters. The modeling process proceeded in two steps; first simple generalized linear regression models were fitted to the data in order to find independent variables with *P* <  0.20. In the second phase, the variables with *P* < 0.20 and the variables with *P* < 0.05 were then used to fit separate multiple generalized linear regression models. We also checked the correlation between different variables before starting multiple generalized linear regression models. Maternal BMI, LDL-C and HOMA-IR were not included due to their high correlation with maternal weight (*r* = 0.91, *p* < 0.001), total cholesterol (*r* = 0.80, *p* < 0.001) and insulin (*r* = 0.98, *p* < 0.001) respectively. At each stage of the model development, the Akaike Information Criteria (AIC) was used to check if the inclusion of a variable or variables improved the model fit [27]. The model with the smallest AIC value was considered as a better fit. All analyses were done using Stata SE 14 and IBM SPSS 24 and the significance level was two sided and set at α = 0.05.

## Results

Table 1 showed the baseline characteristics of the pregnant women based on their BMI measured at their first visit at 10.1 ± 2.2 weeks gestation. Among the 498 women, 154 (30.9%) were underweight, 241 (48.4%) had normal weight and 103 (20.7%) were overweight. The mean age of the women at baseline were 20 ± 2.6 years. As expected, all the anthropometric indices (including weight, BMI, waist, waist-hip ratio, and MUAC), systolic and diastolic blood pressure were significantly lower in underweight women. Underweight mothers (compared with normal weight and overweight counterparts) consumed lower fat, whilst ingesting similar levels of carbohydrates and protein; furthermore, they were more physically active.

**Table 1:** Baseline characteristics of pregnant women based on their BMI (kg/m^2^)

Variables	Overall	Underweight (BMI < 18.5)	Normal weight (BMI 18.5- < 23)	Overweight (BMI ≥23)
Number, %	498	154 (30.9)	241 (48.4)	103 (20.7)
Age (years)	20.0 (2.6)	19.4 (2.0)	19.7 (2.4)	21.5 (3.4) *†
Gestational age (weeks)	10.1 (2.2)	10.2 (2.2)	10.1 (2.3)	9.9 (2.3)
High SES (> 10,000 BDT), %	160 (32.1)	48 (31.2)	81 (33.6)	31 (30.1)
Weight (Kg)	46.1 (8.5)	38.5 (3.3)	46.0 (4.6) *	57.8 (7.7) *†
Height (cm)	149.7 (5.4)	149.7 (5.3)	149.7 (5.5)	149.6 (5.5)
BMI (kg/m^2^)	20.6 (3.4)	17.2 (0.99)	20.5 (1.2) *	25.8 (2.9) *†
Waist (cm)	76.5 (9.2)	68.8 (5.0)	76.5 (5.9) *	88.1 (8.4) *†
Hip (cm)	90.5 (7.9)	83.4 (4.9)	90.7 (4.2) *	100.8 (6.7) *†
WHR	0.85 (0.13)	0.83 (0.07)	0.84 (0.05)	0.90 (0.25) *†
MUAC (cm)	23.8 (2.9)	21.3 (1.4)	24.0 (1.8) *	27.2 (2.9) *†
Systolic BP (mmHg)	92.8 (9.3)	90.2 (8.5)	92.9 (9.0) *	96.4 (10.3) *†
Diastolic BP (mmHg)	63.2 (7.2)	61.4 (6.0)	62.7 (6.9)	67.0 (7.9) *†
Physical inactivity, %	35 (7.0)	3 (1.9)	21 (8.7) *	11 (10.7) *
Energy (Kcal/day)	1489.7 (175.9)	1473.2 (175.6)	1487.2 (173.3)	1520.9 (180.0)
Carbohydrate (gm)	215.0 (35.1)	213.7 (34.5)	214.6 (34.0)	217.6 (38.7)
Protein (gm)	52.3 (13.8)	52.8 (13.6)	51.8 (13.8)	53.0 (14.3)
Fat (gm)	45.8 (9.4)	44.4 (9.4)	46.0 (9.2)	47.8 (8.7) *

Data are presented as mean (SD) or number (%)

*p-value < 0.05 compared with underweight*;* †*p*-value < 0.05 compared with normal. Abbreviation: *BMI* body mass index, *SES* socio-economic condition, *BDT* Bangladeshi Taka (1 UDS = 80 BDT), *WHR* waist-hip ratio, *MUAC* mid upper arm circumference, *BP* blood pressure

Table 2 showed the baseline biochemical parameters of the pregnant women based on their BMI. Significantly lower levels of fasting plasma glucose, 2 h plasma glucose and triglyceride at baseline were observed among underweight compared to overweight mothers (*p* < 0.05). In addition, insulin, HOMA-IR, cholesterol, LDL-C and leptin levels were significantly lower in underweight women than normal weight and overweight women. No significant difference was observed in vitamin D, vitamin B12, homocysteine, folate and ferritin levels between three groups. The prevalence of anemia (Hb, < 11 g/dl), vitamin D deficiency (< 30 nmol/l), vitamin B12 deficiency (< 200 pg/ml), high homocysteine level (> 15 μmol/l), folate deficiency (< 3 ng/ml) and iron deficiency (ferritin < 13 ng/ml) were 19.5, 46.4, 15.1, 1.2, 0.4, and 12.7% respectively. Anemia was significantly higher in underweight women than in women with normal weight (27.3 vs 15.4%, *p* = 0.004). GDM was found in 18.4% women (58 out of 315 at 24–28 weeks). The prevalence of GDM was significantly higher in overweight women (28.1%) than underweight (16.7%) and normal weight women (16.0%: *p* < 0.05). Only three women (0.95%) were diagnosed with diabetes in pregnancy.

**Table 2:** Baseline biochemical parameters of pregnant women based on their BMI (kg/m^2^)

Variables	Overall	Underweight (BMI < 18.5)	Normal weight (BMI 18.5- < 23)	Overweight (BMI ≥23)
Number, %	498	154 (30.9)	241 (48.4)	103 (20.7)
Hb (g/dl)	12.1 (11.3, 12.9)	12.1 (10.9, 12.9)	12.1 (11.3, 12.9)	12.1 (11.3, 13.3)
Anemia (Hb < 11 g/dl), %	97 (19.5)	42 (27.3)	37 (15.4) *	18 (17.5)
FPG (mmol/l)	4.7 (4.5, 5.0)	4.7 (4.5, 4.9)	4.7 (4.5, 5.1)	4.8 (4.6, 5.2) *†
2hPG (mmol/l)	6.2 (5.3, 7.0)	6.3 (5.4, 7.6)	6.4 (5.4, 7.3)	7.0 (6.1, 8.0) *†
GDM, % ^a^	58 (18.4)	16 (16.7)	26 (16.0)	16 (28.1) *†
Insulin (μIU/ml)	7.6 (5.4, 10.3)	7.1 (5.0, 8.9)	7.6 (5.4, 10.0) *	9.7 (7.5, 15.2) *†
HOMA-IR	1.6 (1.1, 2.2)	1.5 (1.0, 1.9)	1.6 (1.1, 2.2) *	2.1 (1.6, 3.2) *†
Cholesterol (mg/dl)	163.0 (150.0, 180.0)	156.0 (150.0, 170.3)	162.0 (150.0, 181.0) *	172.0 (156, 200) *†
Triglycerides (mg/dl)	102.5 (80.0, 132.8)	91.5 (75.0, 124.5)	100.5 (78.0, 136.8)	122.0 (92, 147.0) *†
HDL-C (mg/dl)	43.0 (38.0, 48.8)	43.0 (38.8, 49.0)	42.0 (37.0, 52.0)	42.0 (36.0, 48.0)
LDL-C (mg/dl)	97.0 (88.0, 112.0)	94.0 (84.8, 104.0)	99.0 (90.0, 114.0) *	104.0 (92.0, 125.0) *
Vitamin D (nmol/l)	30.2 (18.6, 41.8)	30.5 (18.1, 42.5)	31.2 (17.7, 43.8)	32.8 (20.3, 47.2)
Deficiency (< 30 nmol/l), %	231 (46.4)	75 (48.7)	112 (46.5)	44 (42.7)
Vitamin B12 (pg/ml)	296.5 (221.5, 389.8)	295.0 (222.3, 432.8)	304.0 (226.3, 406.5)	295.0 (251.3, 370.5)
Deficiency (< 200 pg/ml), %	76 (15.1)	26 (16.9)	40 (16.6)	10 (9.7)
Homocysteine (μmol/l)	7.0 (5.9, 8.8)	7.0 (5.7, 8.8)	7.0 (5.7, 8.6)	6.8 (5.7, 8.1)
High (> 15 μmol/l), %	6 (1.2)	3 (1.9)	3 (1.3)	–
Folate (ng/ml)	10.5 (7.7, 18.5)	11.3 (7.6, 18.7)	10.3 (7.6, 20.1)	9.8 (7.6, 14.3)
Deficiency (< 3 ng/ml), %	2 (0.4)	–	–	2 (1.9)
Ferritin (ng/ml)	45.6 (23.3, 75.2)	45.4 (25.9, 84.2)	45.9 (22.6, 72.7)	49.0 (28.7, 75.6)
Deficiency (< 13 ng/ml), %	63 (12.7)	16 (10.4)	31 (12.9)	16 (15.7)
Leptin (ng/ml)	7.3 (4.6, 12.2)	4.6 (3.4, 6.6)	7.3 (5.4, 11.1) *	16.2 (11.4, 23.2) *†

Data are presented as median (interquartile range) or number (%). Biochemical assays were all measured in serum. ^a^ from 2nd visit

*p-value < 0.05 compared with underweight*;* †*p*-value < 0.05 compared with normal. Abbreviation: *BMI* body mass index, *FPG* fasting plasma glucose, *2hPG* 2 h plasma glucose, *GDM* gestational diabetes, *HOMA-IR* homeostasis model assessment of insulin resistance, *HDL-C* high density lipoprotein cholesterol, *LDL-C* low density lipoprotein cholesterol

As seen in Fig. 138 of 498 mothers were followed-up until the birth of their child. Those mothers lost to follow up differed at their first visit (14–18 weeks) in that they had a lower weight (47.0 vs 45.2 kg; *p* < 0.01), reduced height (151 vs 149 cm; *p* < 001), increased 2 h glucose (6.2 vs 6.8 mmol/l; *p* < 001) and increased vitamin D levels (31 vs 35.3 pg/ml; *p* = 0.01).

Table 3 showed the demographic and biochemical characteristics of neonates by birth weight category. Among the 138 neonates, 16 (11.6%) had LBW (< 2500 g) and only 8 (5.8%) were preterm (< 37 weeks). Mean weight (grams), length (cm), weight for length (kg/m^2^), head circumference (cm), MUAC (cm) and ponderal index was 2814 ± 404, 45.9 ± 2.7, 13.4 ± 1.7, 33.1 ± 1.4, 9.8 ± 0.8 and 2.9 ± 0.5, respectively. LBW babies had significantly lower length, weight for height, head circumference, MUAC and ponderal index.

**Table 3:** Demographic and biochemical characteristics of neonates at birth, categorized by birth weight

Variables	Overall	Low birth weight (weight < 2500 g)	Normal birth weight (weight ≥ 2500 g)
Number, % ^a^	138	16 (11.6)	122 (88.4)
Sex (male), n, % ^a^	74 (53.6)	5 (31.3)	69 (56.6)
Preterm (< 37 weeks), n, % ^a^	8 (5.8)	3 (18.8)	5 (4.1) *
Delivery (caesarian section), n, % ^a^	90 (65.2)	8 (50.0)	82 (67.2)
Gestational age at delivery (weeks) ^b^	40.2 (1.6)	40.4 (2.6)	40.2 (1.4)
Weight (gram) ^b^	2814 (404)	2146 (166)	2899 (342) *
Length (cm) ^b^	45.9 (2.7)	43.6 (2.8)	46.3 (2.5) *
Weight for height (kg/m^2^) ^b^	13.4 (1.7)	11.3 (1.0)	13.6 (1.6) *
Head circumference (cm) ^b^	33.1 (1.4)	31.4 (1.4)	33.3 (1.3) *
MUAC (cm) ^b^	9.8 (0.8)	9.1 (0.7)	9.9 (0.8) *
Ponderal index ^b^	2.9 (0.5)	2.6 (0.4)	3.0 (0.5) *
Biochemical parameters			
Blood glucose (mmol/l) ^c^	3.8 (3.3, 5.1)	4.3 (3.2, 5.1)	3.8 (3.3, 5.1)
Insulin (μIU/ml) ^c^	6.5 (5.0, 11.5)	5.6 (3.5, 15.1)	6.6 (4.6, 11.4)
HOMA-IR ^c^	1.18 (0.74, 2.30)	1.23 (0.56, 2.85)	1.18 (0.74, 2.27)
Glucose to insulin ratio ^c^	10.6 (7.1, 15.6)	10.9 (6.1, 17.9)	10.5 (7.4, 15.2)
Cholesterol (mg/dl) ^c^	66.0 (56.0, 78.0)	64.0 (47.0, 77.0)	66.0 (56.3, 78.8)
Triglycerides (mg/dl) ^c^	35.0 (26.0, 50.0)	48.0 (28.0, 76.0)	34.5 (25.3, 48.0)
HDL-C (mg/dl) ^c^	23.0 (19.0, 30.0)	19.0 (16.0, 29.0)	24.0 (20.0, 30.0)
LDL-C (mg/dl) ^c^	33.0 (25.0, 41.0)	32.0 (17.0, 39.0)	33.0 (25.0, 41.0)
Vitamin D (nmol/l) ^c^	33.9 (28.3, 45.2)	31.2 (27.8, 45.3)	34.5 (28.7, 45.2)
Vitamin B12 (pg/ml) ^c^	417.0 (329.3, 640.3)	414.0 (258.5, 669.0)	421.0 (340.0, 629.5)
Homocysteine (μmol/l) ^c^	5.0 (4.0, 6.4)	5.5 (4.5, 6.9)	5.0 (4.0, 6.3)
Folate (ng/ml) ^c^	16.8 (14.2, 21.8)	13.7 (12.3, 20.3)	16.8 (14.4, 22.2)
Ferritin (ng/ml) ^c^	119.0 (58.8, 197.0)	112.0 (61.1, 155.0)	127.0 (59.3, 200.0)
Leptin (ng/ml) ^c^	5.1 (3.0, 7.8)	5.3 (2.9, 7.1)	5.1 (3.0, 8.1)

Data are presented as ^a^ number (%) or ^b^ mean (SD) or ^c^ median (interquartile range) where appropriate. Biochemical assays were all measured in serum. *p-value < 0.05 compared with LBW*.* Abbreviation: *LBW* low birth weight, *MUAC* mid upper arm circumference, *HOMA-IR* homeostasis model assessment of insulin resistance, *HDL-C* high density lipoprotein cholesterol, *LDL-C* low density lipoprotein cholesterol

No significant difference was observed for any of the following parameters (blood glucose, insulin, HOMA-IR, glucose to insulin ratio, cholesterol, triglyceride, HDL-C, LDL-C, vitamin D, vitamin B12, folate, ferritin, homocysteine and leptin birth weight levels) in LBW babies compared to normal weight infants.

Table 4 showed the anthropometric and laboratory parameters of neonates categorized by their mothers’ BMI. No significant difference for anthropometric indices (birth weight, birth length, head circumference, and MUAC) was observed among neonates born to underweight mothers at inclusion than normal weight mothers. Significant higher cord blood glucose (4.3 vs 3.7 mmol/L), blood cholesterol (67.0 vs 60.5 mg/dl), HDL-C (25.0 vs 21.5 mg/dl) and lower vitamin B12 (372.0 vs 514.5 pg/ml) values were observed in neonates born to underweight mothers than overweight/obese mothers.

**Table 4:** Anthropometric and laboratory parameters of neonates at birth, categorized by maternal BMI (kg/m^2^)

	Underweight (BMI < 18.5)	Normal weight (BMI 18.5- < 23)	Overweight (BMI ≥23)
Total number (*n* = 138)	38	67	33
Anthropometric indices			
Birth weight (gram)^a^	2700 (400)	2800 (400)	2900 (400)
Birth length (cm) ^a^	45.4 (3.1)	46.3 (2.6)	45.9 (2.3)
Head circumference (cm) ^a^	32.9 (1.3)	33.0 (1.5)	33.4 (2.7)
MUAC (cm) ^a^	9.7 (0.8)	9.8 (0.8)	9.8 (0.9)
Laboratory parameters			
Blood glucose (mmol/l) ^b^	4.3 (3.4, 5.2)	3.7 (3.3, 5.1)	3.7 (3.2, 4.2) *
Insulin (μIU/ml) ^b^	6.0 (4.3, 13.5)	7.3 (3.2, 12.1)	6.1 (4.6, 9.4)
HOMA-IR ^b^	1.1 (0.7, 2.7)	1.4 (0.8, 2.6)	1.1 (0.7, 1.5)
Glucose to insulin ratio ^b^	11.5 (8.3, 16.0)	10.0 (6.5, 16.1)	10.3 (7.2, 15.1)
Cholesterol (mg/dl) ^b^	67.0 (58.5, 85.0)	67.5 (56.3, 75.8)	60.5 (50.8, 66.3) *
Triglycerides (mg/dl) ^b^	40.0 (28.5, 48.5)	35.5 (24.0, 52.0)	34.0 (28.0, 53.0)
HDL-C (mg/dl) ^b^	25.0 (20.0, 34.0)	23.0 (19.3, 31.0)	21.5 (17.5, 27.0) *
LDL-C (mg/dl) ^b^	36.0 (25.0, 43.0)	35.5 (26.5, 41.8)	30.0 (24.8, 35.0)
Vitamin D (nmol/l) ^b^	31.7 (27.1, 42.7)	33.6 (28.4, 43.7)	38.2 (31.5, 46.6)
Vitamin B12 (pg/ml) ^b^	372.0 (297.3, 496.8)	424.5 (297.0, 641.5)	514.5 (369.0, 751.0) *†
Homocysteine (μmol/l) ^b^	5.4 (4.6, 7.2)	5.0 (4.0, 6.0)	4.7 (3.6, 6.4)
Folate (ng/ml) ^b^	15.8 (13.2, 24.8)	17.3 (13.8, 22.4)	16.5 (14.5, 18.7)
Ferritin (ng/ml) ^b^	103.8 (61.5, 253.8)	142.5 (64.4, 203.8)	81.6 (43.5, 161.0)
Leptin (ng/ml) ^b^	4.3 (2.7, 8.1)	5.1 (2.9, 8.8)	5.7 (4.0, 7.1)

Data are presented as ^a^ mean (SD) or ^b^ median (interquartile range)

*p-value < 0.05 compared with underweight*;* †*p*-value < 0.05 compared with normal. Abbreviation: *BMI* body mass index, *MUAC* mid upper arm circumference, *HOMA-IR* homeostasis model assessment of insulin resistance, *HDL-C* high density lipoprotein cholesterol, *LDL-C* low density lipoprotein cholesterol

Table 5 showed the associations between neonatal birth weight and maternal characteristics in early pregnancy. Maternal height and weight at baseline were positively associated with neonatal birth weight in the univariate analysis. In the adjusted analysis (model 2), maternal height was positively associated with neonatal birth weight (*p* = 0.02) while homocysteine was negatively associated with neonatal birth weight (*p* = 0.02). Model 2 showed that a unit increase in homocysteine was associated with a 33.26 g decrease in mean birth weight (*p* = 0.02).

**Table 5:** Generalized linear regression analysis showing the associations between neonatal birth weight and maternal measures. Using the Akaike information criteria (AIC), model 2 was selected because it has the smallest AIC value

Maternal variables	Univariate (unadjusted)		^1^Full model (*n* = 138) AIC = 2071.32		^2^Model 2 (*n* = 138) AIC = 2053.40		^3^Model 3 (*n* = 138) AIC = 2066.53	
	β (95% CI)	*P*-value	β (95% CI)	*P*-value	β (95% CI)	*P*-value	β (95% CI)	*P*-value
Height (cm)	17.42 (5.82, 29.01)	< 0.01	20.32 (5.65, 34.99)	0.01	14.97 (2.51, 27.43)	0.02	13.1 (0.76, 25.42)	0.04
Weight (kg)	11.15 (3.20, 19.09)	0.01	3.80 (−6.84, 14.44)	0.48	6.86 (−1.62, 15.35)	0.11	7.75 (−0.67, 16.16)	0.07
Homocysteine (μmol/l)	−26.67 (−54.78, 1.44)	0.06	−32.99 (− 62.03, −3.95)	0.03	−33.26 (− 60.21, − 6.31)	0.02		
2hPG (mmol/l)	44.93 (− 15.76, 105.62)	0.15	51.54 (− 11.97, 115.06)	0.11	51.31 (−8.00, 110.63)	0.09		
Triglycerides (mg/dl)	−0.93 (−2.21, 0.34)	0.15	−0.96 (−2.48, 0.56)	0.22	−1.09 (− 2.31, 0.14)	0.08		
Hemoglobin	−24.88 (− 80.13, 30.38)	0.38	−5.75 (−68.68, 57.18)	0.86				
FPG (mmol/l)	70.77 (−70.28, 211.82)	0.32	46.92 (−97.80, 191.65)	0.53				
Insulin (μIU/ml)	7.38 (−6.76, 21.51)	0.30	11.07 (−4.59, 26.74)	0.17				
Cholesterol (mg/dl)	−0.43 (−3.30, 2.44)	0.77	−0.94 (− 4.36, 2.48)	0.59				
HDL-C (mg/dl)	5.59 (−4.88, 16.06)	0.29	6.01 (−5.43, 17.44)	0.30				
Vitamin D (nmol/l)	−0.96 (− 5.27, 3.35)	0.66	−0.45 (− 5.01, 4.11)	0.85				
Vitamin B12 (pg/ml)	0.10 (−0.40, 0.60)	0.69	−0.07 (− 0.60, 0.46)	0.80				
Ferritin (ng/ml)	0.56 (−0.98, 2.11)	0.47	0.45 (−1.15, 2.04)	0.58				
Folate (ng/ml)	−2.72 (−10.88, 5.43)	0.51	−0.51 (−9.00, 7.97)	0.91				
Age (years)	12.69 (−10.50, 35.88)	0.28	−6.08 (−32.70, 20.55)	0.66				
Gestational age in weeks	−11.83 (−55.64, 31.98)	0.60	−24.20 (−70.14, 21.74)	0.30				
BMI (kg/m^2^)	16.28 (−3.01, 35.58)	0.10						
LDL-C (mg/dl)	−0.27 (−3.29, 2.75)	0.86						
HOMA-IR	41.48 (−18.76, 101.72)	0.18						

^1^Model 1 is adjusted for all maternal variables ^2^Model 2 was fitted by selecting variables with *P* ≤ 0.2 from the univariate analysis

^3^Model 3 was fitted by selecting variables with *P* ≤ 0.05 from the univariate analysis

Abbreviation: *2hPG* 2 h plasma glucose, *FPG* fasting plasma glucose, *HDL-C* high density lipoprotein cholesterol, *BMI* body mass index, *LDL-C* low density lipoprotein cholesterol, *HOMA-IR* homeostasis model assessment of insulin resistance

The associations between neonatal birth weight and neonatal cardiometabolic and micronutrients characteristics were presented in Table 6. The univariate analysis showed that folate was positively associated with neonatal birth weight while triglyceride was negatively associated with neonatal birth weight. In the adjusted analysis (model 2), a unit increase in triglycerides was associated with a decrease in birth weight of 2.81 g (*p* = 0.02). However, a unit increase in folate significantly increase birth weight by 5.26 g (*p* = 0.03).

**Table 6:** Generalized linear regression analysis showing the associations between neonatal birth weight and neonatal cardiometabolic and micronutrient variables. Using the Akaike information criteria (AIC), model 2 was selected because it has the smallest AIC value

Neonatal variables	Univariate (unadjusted)		^1^Full Model (*n* = 138) AIC = 1903.11		^2^Model 2 (*n* = 138) AIC = 1713.23		^3^Model 3 (*n* = 138) AIC = 1810.955	
	β (95% CI)	*P*-value	β (95% CI)	*P*-value	β (95% CI)	*P*-value	β (95% CI)	*P*-value
Triglyceride (mg/dl)	−3.60 (−6.11, −1.08)	0.01	−2.18 (−5.27, 0.91)	0.17	−2.81 (− 5.33, −0.29)	0.03	− 3.25 (− 5.75, − 0.75)	0.01
Folate (ng/ml)	5.15 (0.72, 9.57)	0.02	5.41 (0.23, 10.59)	0.04	5.26 (0.47, 10.05)	0.03	4.77 (0.45, 9.09)	0.03
Blood glucose (mmol/l)	−41.98 (−88.39, 4.43)	0.08	−29.54 (−97.25, 38.16)	0.39	−38.39 (−90.27, 13.50)	0.15		
Glucose insulin ratio (GIR)	−5.38 (−12.60, 1.84)	0.14	−2.98 (−16.77, 10.81)	0.67	−0.28 (− 8.66, 8.10)	0.95		
Homocysteine (μmol/l)	−27.39 (−59.44, 4.67)	0.09	−21.43 (−55.87, 13.01)	0.22	−18.34 (− 50.38, 13.70)	0.26		
Insulin (μIU/ml)	3.04 (−8.85, 14.94)	0.61	−8.32 (−29.48, 12.83)	0.44				
Cholesterol (mg/dl)	−1.62 (−4.99, 1.74)	0.34	−6.15 (−13.55, 1.25)	0.10				
HDL- C (mg/dl)	3.18 (−3.83, 10.19)	0.37	9.91 (−1.52, 21.34)	0.09				
LDL-C (mg/dl)	0.06 (−4.92, 5.04)	0.98	8.57 (−0.79, 17.92)	0.07				
Vitamin D (nmol/l)	1.10 (−4.41, 6.61)	0.69	1.45 (−4.01, 6.91)	0.60				
Vitamin B12 (pg/ml)	0.02 (−0.30, 0.35)	0.89	−0.03 (− 0.38, 0.33)	0.87				
Ferritin (ng/ml)	0.06 (−0.62, 0.74)	0.86	−0.06 (− 0.77, 0.65)	0.87				
Leptin (ng/ml)	4.81 (−9.24, 18.87)	0.50	−0.75 (−14.71, 13.20)	0.92				
Gestational age in weeks	−11.83 (−55.64, 31.98)	0.60	− 19.19 (−68.13, 29.74)	0.44				

^1^Model 1 is adjusted for all neonatal variables ^2^Model 2 was fitted by selecting variables with *P* ≤ 0.2 from the univariate analysis

^3^Model 3 was fitted by selecting variables with *P* ≤ 0.05 from the univariate analysis

Abbreviation: *HDL-C* high density lipoprotein cholesterol, *LDL-C* low density lipoprotein cholesterol

**Fig. 2:**
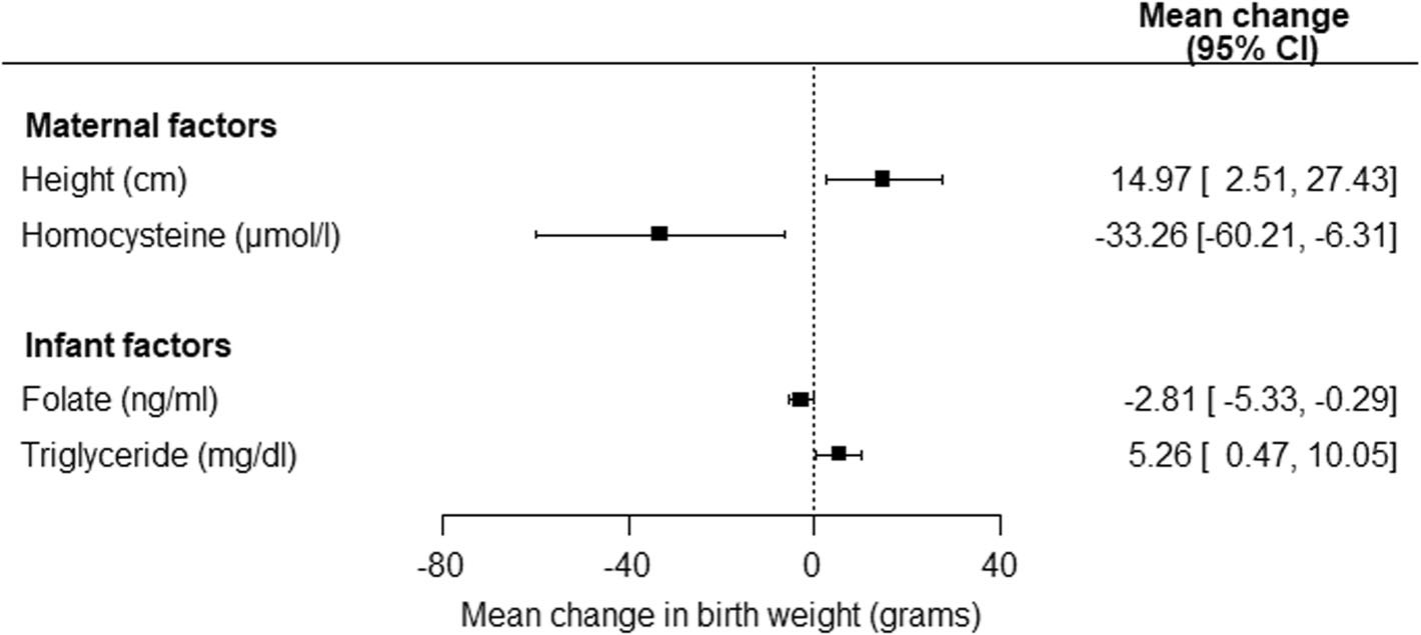
Maternal and neonatal factors that were significantly associated with neonatal birth weight

Figure 2 illustrated maternal (including height and homocysteine) and neonatal (including cord triglyceride and folate) factors that were significantly associated with neonatal birth weight.

## Discussion

Our data reports the maternal characteristics and nutritional status in early pregnancy (6–14 weeks) and its impact on neonatal birth weight. High prevalence of both underweight (< 18.5 kg/m^2^) and overweight (BMI ≥23 kg/m^2^) were observed in our study. Our findings are in line with 2007 Bangladesh Demographic and Health Survey (BDHS) [28]. As our assessment occurred in early pregnancy, the observed weight gain is likely related to the pre-pregnancy status. Our findings are consistent with other rural and urban studies in Bangladesh who also identified similar prevalence of women with low BMI in early pregnancy, but the rate of overweight/obesity was lower. This is likely to be a consequence of applying the newer WHO defined cutoff levels for BMI in South Asian populations [29–31].

In our study, we assessed the level of micronutrient status in early pregnancy (gestational age 10.1 ± 2.2 week), therefore the chances of confounding effect of hemodilution which is common in late pregnancy was unlikely. Our data showed a high prevalence of vitamin D deficiency and insufficiency in pregnant women in early pregnancy. Studies in Pakistan with identical definition for vitamin D deficiency with similar inclusion criteria had shown similar results [15, 32]. Similar findings were also observed in other studies conducted in Bangladesh [33] and India [34].

Limited dietary sources, dark skin pigmentation, and religious or cultural practices that limit skin exposure to sunlight may prevent endogenous production of vitamin D, as illustrated by El-Sonbaty and Abdul-Ghaffar et al. [35] and Guzel et al. [36] may in part explain the higher occurrence of hypovitaminosis in a population with abundant exposure to sunlight year-round. The average sun exposure in Dhaka was 12 h and above during the months of recruitment (April – September) and therefore it is less likely that vitamin D levels may have been impacted by sunlight exposure. Most importantly daily dietary intake of vitamin D was low (normal 15 μgm vs 4.34 μgm) in our study population. This may suggest that dietary intervention with vitamin D rich foods may be an appropriate strategy to address the issue.

Increasing evidence suggest that vitamin B12 deficiency is highly prevalent in women of reproductive age, particularly amongst populations with limited intake of animal source foods [32, 37]. Evidence also suggests that vitamin B12 concentration gradually decline throughout gestation, therefore, it is challenging to assess the prevalence of deficiency in pregnant women. Studies have shown that based on gestational week, prevalence of vitamin B12 deficiency may vary from 5% (< 28 days gestation) to 72% (immediately prior to delivery) worldwide [38]. The JiVitA study in rural north-western Bangladesh found a prevalence of 20% deficiency in early pregnancy [39]. Another urban study conducted in similar settings of present study in Dhaka city found 26% deficiency in early pregnancy [40]. Our finding is in line with national micronutrient status survey in Bangladesh [41] and Amsterdam Born Children and their Development (ABCD) study, Netherland [42]. However, our results are in contrast with Pune, Indian study [37] and Maternal and Infant Nutrition Interventions in Matlab (MINIMAT) trial in Bangladesh where reported B12 status (46% deficiency) early in their third trimester [31]. One-third of women in Pune study were vegetarian and they consumed less protein (44.9 vs 52.3 g) and fat (32.7 vs 45.8 g) than our participated women. However, average daily dietary intake of vitamin B12 in our study population need to be increased as this was observed low (1.62 mcg) compare to recommended daily intake (2.6 mcg) for Bangladeshi population. Further, Bangladesh diet contains high level of fish compared to many societies with non-vegetarian diet.

Regarding iron and folate deficiency, our findings are consistent with other previous urban study in Bangladesh [40]. Lower levels of folate deficiency (0.2%) was also observed in Pune, India study. Further, it was reported that higher maternal folate concentrations predict greater adiposity and higher insulin resistance in neonates [43]. Furthermore, universal distribution of folate and iron to all pregnant mothers in Bangladesh may have reduced folate deficiency.

The prevalence of GDM in our study was 18.4%. Two similar studies in India and China with identical definition for GDM reported 18.5 and 19.9% prevalence respectively [44, 45]. Bangladesh still has high maternal mortality rate (340/100,000), which is one of the highest in the world [46]. It is alarming though to observe the higher occurrence of GDM in women defined as under or normal weight, considered as low risk. Further investigation is required to define the disposition of the phenotype and prognosis particularly at a time when the upswing of diabetes is threatening the health system in poor countries.

Like many other developing countries, millions of Bangladeshi children suffer from nutritional deficiencies due to their mothers’ poor nutritional status prior to and during pregnancy. Children are often born with LBW. According to National LBW Survey 2003–2004 reports, 36% babies were born with LBW. The mean birth weight of infants in Bangladesh is 2632 g and the mean birth length is 48.5 cm [47]. In our study, incidence of LBW and preterm delivery were 11.6 and 5.8% respectively in our study. Preterm birth rate found higher in LBW babies. Bangladesh Demographic and Health Survey (BDHS) 2011 and MINIMAT trail reported 16 and 20% LBW respectively [41, 31]. They had significantly lower head circumference but there was no significant difference in gestation age or with mother’s BMI status in early pregnancy.

Our results showed that neonates born to underweight mothers had significantly higher blood glucose, higher cholesterol, but higher HDL-C, and lower vitamin B12 than neonates born to overweight mothers irrespective of their birth weight. Mechanisms underlying these associations are still poorly understood. However, our findings reflect the possible interaction of environmental factors and fetal growth and the in-utero glucose and lipid metabolism. Future long-term longitudinal studies in different ethnicities would help to explore the underlying mechanisms.

Finally, we investigated both maternal and neonatal nutritional and cardiometabolic factors on newborn birth weight. In our study, maternal height and weight at inclusion were positively associated with neonatal birth weight in univariate analysis. In the adjusted analysis, maternal height was positively, and homocysteine was negatively associated with neonatal birth weight. A series of studies investigated the relationship between maternal height and birth weight showed that shorter maternal height was associated with reduced fetal growth and low birth weight [31, 48–51]. They concluded that the primary reason for this association was undernutrition/malnutrition. Negative association of maternal homocysteine concentrations with newborn birth weight is consistent with studies conducted in India [52, 53] and Norway [54]. We observed a unit increase in homocysteine was associated with a 33.3 g decrease in mean birth weight in our study. Interestingly, a similar finding has also been observed in a systemic review and meta-analysis conducted by Hogeveen M et al [55] They reported that a one standard deviation (SD) increase in maternal homocysteine corresponded to a decrease in birth weight of 31 g. Homocysteine is a well-recognized risk factor for endothelial dysfunction and cardiovascular disease. Homocysteine concentrations are influenced by vitamin B status, mainly by folate and Vit B12. Therefore, vitamin B supplementation should be introduced to the pregnant women for lowering maternal homocysteine level and reducing its wide range of adverse pregnancy outcomes, including recurrent early pregnancy loss, preeclampsia, LBW, fetal loss and future risk of cardiovascular disease.

Among the neonatal variable, cord folate was positively, and triglyceride was negatively associated with birth weight. Literature has shown that folic acid supplement during the last 12 to 16 weeks of pregnancy delivered heavier babies than those born to mothers receiving iron alone. Further, it was found that the effect of the folic acid supplements was more marked on first born children [ 56].

As in other studies, we have found that lower birth weight was associated with elevated cord triglyceride concentrations [57–59]. Reduced lipoprotein lipase (LPL) activity might be the theoretical explanation for the inverse correlation between fetal cord triglyceride and birth weight. It is known that the expression of LPL is strongly associated with the development of adipose tissue. LBW infants appear to have impaired utilization of circulating triglyceride, because of lacking peripheral adipose tissue [60].

Among the strengths of the study were interviewer training for interview technique, questionnaires, recall of the participants, anthropometric measurements, collection of blood, and its subsequent processing (centrifugation and storage). Primigravida mothers were recruited in their 1st trimester to avoid any noise interfering with the analysis. All the maternal and cord blood parameters were determined in the same laboratory, should reduce the chance of error due to different analysis technique. This study also creates an opportunity for long time follow-up of study participants and their offspring which will serve as a relevant data bank for future studies. The limitations of the present study include a higher number of dropouts and thereby limited number of neonatal samples especially those with very low birthweight (*n* = 16). It should be noted that the exclusion of GDM mothers in their second visit, political violence during the study period were the major causes of participants’ dropout. In addition, the local cultural practice in Bangladesh is that primigravida mothers deliver the child in their parent’s house; this caused a major undertaking to trace all the participants. Because of limited project budget funds and the logistics of cord blood collection and sample processing, we could not collect samples from the majority of home deliveries; analysis of the neonates was therefore mainly restricted to hospital deliveries to ensure data integrity. Reassuringly, with respect to analysis of the neonate data, there is no significant difference in terms of the nutrition status in those mothers who were recruited initially (*n* = 498) and those who delivered in the hospital (*n* = 138). At recruitment, 30.9% of the women were categorized as having low BMI (< 18.5), 48.4% normal BMI (18.5–22.9), and 23.9% (overweight and obese). The samples from the newborns were collected from (n = 138) deliveries from the hospitals. Those mothers who delivered were categorized as low BMI were 27.5%, normal 48.6% and over-weight and obese were 23.7%. The difference was not statistically significant (*p* = 0.43). Therefore, it is less likely that the results were influenced by the high rate of dropouts.

Even though areas of residence were selected based on a random sampling procedure but the population were selected from five areas in and around Dhaka city. The people in Bangladesh (98–99%) belong to one ethnic group and have similar body structure. Therefore, results may be interpreted with caution to the Indian Bengali population.

However, we could not find any significant differences in the baseline-data for mothers delivered in the hospital or home related to BMI, and therefore, it is less likely to have impacted the results. Home delivery is common in Bangladesh especially in rural and semi-urban population [40]. Micronutrients were assessed only at early gestation in this study, whereas they might also be important for fetal growth at the later gestational period [7–9]. This could be another potential limitation of this study.

## Conclusion

Maternal malnutrition including both micro (iron deficiency, vitamin D deficiency, vitamin B12 deficiency, high homocysteine level and folate deficiency) and undernutrition as measured by BMI was highly prevalent in early pregnancy. LBW was associated with lower folate, elevated cord triglyceride concentrations of the neonates and mother’s height and increase in maternal homocysteine levels. Neonates born to underweight mothers exhibited significantly higher blood glucose, cholesterol, higher HDL-C and lower vitamin B12 irrespective of their birth weight although this conclusion should be tempered by the numbers studied. The results may call for closer monitoring of pregnant women by the antenatal clinics. The data has also important implications for pregnancy care in Bangladesh and other similar communities.

## Data Availability

The datasets during and / or analyzed during the current study available from the corresponding author on reasonable request.

## Abbreviations

AIC: Akaike Information Criteria
ANOVA: Analysis of variance
BDT: Bangladeshi Taka
BIRDEM: Bangladesh Institute of Research and Rehabilitation in Diabetes, Endocrine and Metabolic Disorders
BMI: Body mass index
BP: Blood pressure
CAD: Coronary artery disease
FFQs: Food Frequency Questionnaires
FPG: Fasting plasma glucose
FWV: Family Welfare Visitors
GDM: Gestational diabetes
GIFTS: Genomic and lifestyle predictors of fetal outcome relevant to diabetes and obesity and their relevance to prevention strategies in South Asian peoples
HDL-C: High density lipoprotein cholesterol
HOMA-IR: Homeostasis model assessment of insulin resistance
HTN: Hypertension
2hPG: 2 h plasma glucose
IADPSG: International Association of Diabetes and Pregnancy Study Group
IUGR: Intrauterine growth restriction
T2DM: Type 2 diabetes
LBW: Low birth weight
LDL-C: low density lipoprotein cholesterol
MCHTI: Maternal and Child Health Training Institute
MUAC: Mid upper arm circumference
SES: Socio-economic condition
WHR: Waist-hip ratio
